# Infrared Spectroscopy and Chemometric Applications for the Qualitative and Quantitative Investigation of Grapevine Organs

**DOI:** 10.3389/fpls.2021.723247

**Published:** 2021-09-03

**Authors:** Elizma van Wyngaard, Erna Blancquaert, Hélène Nieuwoudt, Jose Luis Aleixandre-Tudo

**Affiliations:** ^1^South African Grape and Wine Research Institute (SAGWRI), Department of Viticulture and Oenology, Stellenbosch University, Stellenbosch, South Africa; ^2^Instituto de Ingeniería de Alimentos para el Desarrollo (IIAD), Departamento de Tecnologia de Alimentos, Universidad Politécnica de Valencia, Valencia, Spain

**Keywords:** infrared spectroscopy, chemometrics, grapevine organs, non-invasive, carbohydrates, nitrogen, amino acids

## Abstract

The fourth agricultural revolution is leading us into a time of using data science as a tool to implement precision viticulture. Infrared spectroscopy provides the means for rapid and large-scale data collection to achieve this goal. The non-invasive applications of infrared spectroscopy in grapevines are still in its infancy, but recent studies have reported its feasibility. This review examines near infrared and mid infrared spectroscopy for the qualitative and quantitative investigation of intact grapevine organs. Qualitative applications, with the focus on using spectral data for categorization purposes, is discussed. The quantitative applications discussed in this review focuses on the methods associated with carbohydrates, nitrogen, and amino acids, using both invasive and non-invasive means of sample measurement. Few studies have investigated the use of infrared spectroscopy for the direct measurement of intact, fresh, and unfrozen grapevine organs such as berries or leaves, and these studies are examined in depth. The chemometric procedures associated with qualitative and quantitative infrared techniques are discussed, followed by the critical evaluation of the future prospects that could be expected in the field.

## Introduction

The agricultural sector is entering its fourth revolution, thus moving toward more sustainable farming practices. Improved sustainability is achieved through the implementation of digital technologies and precision farming or precision viticulture (Lopo et al., 2015). The practice of precision viticulture can be used to successfully manage the challenges of global competition, decreasing natural resources, and increasing environmental pressures (De Orduña, 2010; Fraga et al., 2012). This practice uses innovative technologies and data science, of which infrared spectroscopy is an example.

The application of infrared technologies in the agricultural sector can supply the means to measure intact, fresh, and unfrozen samples directly, and implement precision viticulture successfully. Infrared spectroscopy is becoming increasingly popular in the agricultural and the agri-food industries for its capability to supply rapid results while remaining cost-effective (Lopo et al., 2015; Diago et al., 2018; Cuq et al., 2020). Infrared spectroscopy provides an invaluable tool for qualitative and quantitative applications in plant and food production. This review article will present an overview of infrared spectroscopy applications, focusing on near infrared (NIR) and mid infrared (MIR) spectroscopy, (i) qualitative investigation of grapevine organs will be examined. Then, (ii) discussing the quantification of specifically carbohydrates, nitrogen, and amino acids using infrared technologies, (iii) assessing the chemometric applications used for qualitative and quantitative analysis and associated performance evaluation indices, and (iv) concluding with future prospects anticipated in the field of infrared spectroscopy applications for viticultural investigations.

## Qualitative Spectral Investigation of Grapevine Organs

Infrared spectroscopy provides information on multiple properties of a sample simultaneously and therefore is considered a fingerprinting technique (Cozzolino, 2014; Dos Santos et al., 2017). When looking at complex organic samples, this spectral fingerprint subjected to chemometric methods can be used to investigate and elucidate compositional characteristics in the sample, as well as the relationship between several metabolites in the plant. Additionally, important information about the similarities or dissimilarities of groups can emerge (Cozzolino et al., 2011; Dos Santos et al., 2017). Thus, infrared spectroscopy technologies are a valuable tool for data acquisition.

A study conducted by Lopo et al. (2015) used a portable NIR instrument to scan fresh soil and leaf samples to identify soil type. Using principal component analysis (PCA) and supervised partial least squares discriminant analysis (PLS-DA) the authors grouped the soil samples according to soil type. The leaf samples showed similar separation which correlated to soil type and indicated the feasibility of scanning fresh leaves for soil type determination. PCA was used to extract groupings while PLS-DA was used for model development to determine soil type (Lopo et al., 2015). This study indicated the benefit of using infrared spectroscopy for the direct measurement of fresh grapevine material.

Musingarabwi et al. (2016) studied Sauvignon blanc berry samples at five distinct phenological stages namely green, pre-véraison, véraison, post-véraison, and ripe using NIR and MIR spectroscopy. Berry samples were analyzed as fresh and frozen, and homogenized to a pulp. The best separation between phenological stages was seen using PCA for fresh homogenized samples measured with MIR. Additionally, separation was also seen using orthogonal PLS-DA for NIR and MIR data. This study linked the absorption bands associated with sugars and organic acids to the separation seen between phenological stages. However, there was some overlapping of certain phenological stages, specifically post-véraison and ripe, that showed some degree of similarity between these stages. Although homogenized samples were used in this study, the fresh samples showed the best separation (Musingarabwi et al., 2016). The results showed that using fresh samples could be beneficial and valuable. This study also demonstrated the capability of infrared spectroscopy to detect variability throughout the growing season.

Furthermore, Dos Santos Costa et al. (2019) investigated the development of classification models for three maturation stages for Shiraz and Cabernet Sauvignon berries. Whole berries were scanned throughout the growing season using visible/near (Vis-NIR) infrared spectroscopy. PCA was used to identify the clustering of the samples based on maturation stages. Although some overlapping was seen, three stages were identified namely green, véraison, and ripe. Once these clusters were identified, supervised PLS—DA models were compiled for classification of grapes according to maturation stage. Maturation stages could be successfully predicted with 93.15% accuracy (Dos Santos Costa et al., 2019). These results not only emphasized that infrared spectroscopy can detect changes occurring during the growing season but also that these changes could be predicted and monitored.

Additionally, Cuq et al. (2020) sampled fresh berries at two phenological stages, pea-size and véraison. Fresh leaves were also sampled at véraison and separated into leaf blades and leaf petioles. PCA was performed on the data and clear separation was seen between the four sample groups. The PCA loadings showed spectral regions that discriminated between the grapevine organs. The same regions were identified when the spectra of each organ was averaged and compared directly (Cuq et al., 2020). Although the results showed that spectral regions could be used to distinguish between organs, the wavenumbers or regions associated with each organ type were not specified.

Two studies measuring dried and ground grapevine samples used PCA and found separation based on grapevine organs (Schmidtke et al., 2012; De Bei et al., 2017). Schmidtke et al. (2012) used MIR and identified separation between trunk and root samples. Separation along PC1 positively correlated to regions from 1,650 to 1,550 cm^–1^ with smaller contributions from 1,500 to 1,300 cm^–1^, while PC2 corresponded with the region between 1,000 and 875 cm^–1^. De Bei et al. (2017) used NIR spectroscopy and noted that the spectra looked similar for trunk and leaf samples with prominent peaks at 4,300, 5,200, and 7,000 but that leaf samples had an additional peak at 5,900 cm^–1^. Although certain wavenumbers associated with grapevine organ groupings were identified in these studies, multiple wavenumbers and regions need to be examined in future research.

A study using Vis-NIR investigated the spectra and response of basal, young, and apical leaves on iron deficiency in young rootstock vines (Rustioni et al., 2017). The spectra were transformed, normalized, and compared directly. The leaves had responded differently to the iron deficiency and these differences were evident in the spectra. Not only were differences seen between leaves from the same shoot, but differences between the veins and interveinal area of a specific leaf were also observed. These differences were mostly based on the chlorophyll synthesis and pigmentation distribution in the leaves (Rustioni et al., 2017). The results indicated that spectroscopy is able to detect subtle differences between leaves of the same shoot and even variation within one leaf.

Recently, non-linear methods were also used for classification purposes (Fuentes et al., 2018; Murru et al., 2019). Grape samples have been classified according to variety and ripeness using Fourier transform infrared spectroscopy (FTIR) and artificial neural networks (ANN). The specific compounds influencing the classification were identified (Murru et al., 2019). Machine learning algorithms together with NIR spectra of grapevine leaves were utilized to compile ANN models for cultivar classification. Classification with 92% accuracy was achieved leading to enhanced capabilities for ampelography (Fuentes et al., 2018). Both linear and non-linear methods need to be considered in future spectral investigations of grapevine organs for increased knowledge.

The studies discussed in this section have reported separation between grapevine organs or phenological stages (Schmidtke et al., 2012; Musingarabwi et al., 2016; De Bei et al., 2017; Dos Santos Costa et al., 2019; Cuq et al., 2020). However, the reasons for the differences in spectral properties observed for the grapevine organs or phenological stages were not fully investigated and could have provided interesting insights. Numerous changes occur in grapevine organs throughout the growing season and lead to large heterogeneity between organs and phenological stages (Hunter et al., 1995; Zapata et al., 2004; Holzapfel et al., 2010; Rossouw et al., 2017). Infrared spectroscopy could be used to monitor changes throughout the growing season and link the changes to specific spectral regions or wavenumbers of interest. Infrared spectroscopy could also be used to identify the characteristic spectral properties of grapevine organs at different phenological stages. Currently, available literature shows a lack of interpretation of the spectral differences perceived for various grapevine organs or phenological stages. More research is required to investigate the reason for the changes in spectral properties throughout the growing season.

## Spectroscopy Techniques for Quantification of Carbohydrates, Nitrogen, and Amino Acids in Grapevines

Although the studies discussed above reported qualitative methods to investigate grapevine organs, most of the same studies focused on quantification methods (Schmidtke et al., 2012; De Bei et al., 2017; Cuq et al., 2020). The following sections focus on the quantification of carbohydrates, nitrogen, and amino acids, henceforth referred to as key metabolites, using infrared spectroscopy methods. Firstly, the importance and role of key metabolites will be discussed. Next, the non-direct approaches will be evaluated, followed by the proposed methods using direct, non-invasive measurement of fresh grapevine material.

### Importance of Carbohydrates, Nitrogen, and Amino Acid Analysis in Grapevines

**FIGURE 1:**
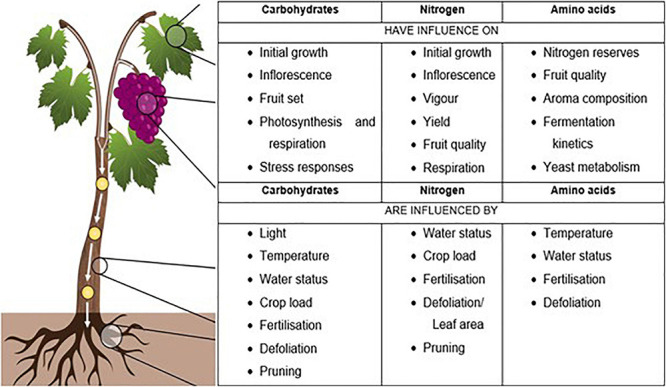
Summary of viticultural, physiological, environmental, and oenological factors that have an influence on, or are influenced by the key metabolites [Adapted from Noronha et al. (2018)].

Carbohydrates, nitrogen, and amino acids form a key part of grapevine physiology affecting growth, yield, and grape quality (Holzapfel et al., 2010; Rossouw et al., 2017). The grapevine source-sink balance causes the concentration of these metabolites to continuously change throughout the growing season (Hunter et al., 1995; Zapata et al., 2004; Rossouw et al., 2017). Carbohydrate and nitrogen reserves play an integral role in vegetative growth and fruiting responses, and are influenced by various factors (Schmidtke et al., 2012; Li-Mallet et al., 2016; Rossouw et al., 2017). The amino acid composition of grape must affects fermentation kinetics, yeast metabolism, and aroma composition. Thus, amino acid content contributes directly to wine quality (Fernández-Novales et al., 2019). Figure 1 summarizes the numerous factors that contribute to carbohydrate, nitrogen, and amino acid concentrations in grapevines.

Despite the importance of these key metabolites, the current analysis methods are costly and time-consuming (Edwards et al., 2011; López et al., 2012; Schmidtke et al., 2012). Using wet chemistry methods to monitor compounds continuously throughout the growing season is not yet feasible. By the time the results are obtained with these methods, concentrations in the grapevine would have changed. The measurement of the key metabolites during the growing season in berries, shoots and leaves could provide valuable information to aid fertilization, irrigation, canopy management, and winemaking decisions (Fernández-Novales et al., 2019; Cuq et al., 2020). New methods for quantifying these metabolites need to be investigated to increase our knowledge, facilitate management decisions, and implement precision viticultural practices.

### Non-direct Quantification Using Infrared Spectroscopy

The quantification of carbohydrate and nitrogen reserves using infrared spectroscopy with non-direct methods was investigated (Schmidtke et al., 2012; Smith et al., 2014; De Bei et al., 2017; Jones et al., 2020). Non-direct methods still use destructive sampling where samples need to be removed in the field and transported to laboratories. The methods also use extensive sample preparation, where the sample material needs to be freeze-dried to remove moisture, and then ground to a powder to obtain a completely homogenized sample. Favorable results were shown for numerous infrared spectroscopy applications and multivariate regression techniques to quantify chemical components in grapevine samples (Schmidtke et al., 2012; Smith et al., 2014; De Bei et al., 2017; Jones et al., 2020).

The use of attenuated total reflectance Fourier transform infrared spectroscopy (ATR-FT-IR) was investigated to assess nitrogen and starch reserves in grapevine wood and root samples (Schmidtke et al., 2012). Over a thousand samples were selected spanning over four vintages, two countries, five locations, and three cultivars, leading to a suitably representative dataset. Cross validation was used with a data split of ten for the calibration subsets. Accurate models were obtained with PLS regression and support vector machine (SVM) regression using dried and powdered samples (Schmidtke et al., 2012). Although sample preparation was still used, the improvement from the existing wet chemistry methods was substantial. This research demonstrated that infrared spectroscopy could be used to develop accurate calibrations for complex compounds across various vintages and locations. Both linear and non-linear regression techniques performed equally well with SVM regression leading to marginally improved models. This study also showed the diverse applications of infrared spectroscopy when sufficient variability is included during the model development stage (Schmidtke et al., 2012).

A related study used NIR spectroscopy to assess non-structural carbohydrates in grapevine trunks and leaves (De Bei et al., 2017). Models were obtained for trunk and leaf samples separately for starch, sugar, and total non-structural carbohydrates using PLS regression. Test set validation was used with the calibration set corresponding to two thirds of the dataset, and validation consisting of one third. Sampling was performed at three phenological stages and clear separation was seen between the phenological stages in the trunk samples’ PCA plot. However, during model development the data for all the stages were combined, although separate models were developed for trunk and leaf samples. The study reported that the models could not provide quantifiable data but that the practical viticultural applications were still significant. The sample set for this study was limited with only 261 trunk samples and 222 leaf samples included. The samples were all collected from one cultivar and vineyard on which irrigation treatments were tested (De Bei et al., 2017). In future research several cultivars and locations could be investigated to increase dataset representativeness as well as model variability and accuracy. Although a limited number of samples could be used to compile sufficient models, special care needs to be taken to ensure that the samples account for the largest possible variability and are representative. Additionally, the irrigation treatment could have negatively influenced the model performance.

A recent study investigated the feasibility of using NIR reflectance spectroscopy to quantify starch in ground and intact grapevine cane samples (Jones et al., 2020). Samples were collected during dormancy and first measured directly with and without bark, and then freeze-dried and ground. Direct measurement led to poor calibration models with no substantial improvement after bark removal (Jones et al., 2020). The calibrations using the ground samples yielded accurate results in agreement with previous studies (Schmidtke et al., 2012; De Bei et al., 2017; Jones et al., 2020).

As discussed, various authors have investigated infrared spectroscopy for the quantification of carbohydrates and nitrogen in grapevine plant material (Schmidtke et al., 2012; De Bei et al., 2017; Jones et al., 2020). However, extensive sample preparation methods were still employed. Most sample preparations change the anatomical and physical properties of the sample. Even fixing the samples in resin could lead to structural and chemical changes that could alter the infrared spectra. Thus, it is suggested that using fresh samples could be less invasive, preserving the sample integrity and structure (Türker-Kaya and Huck, 2017). Current research suggests that methods consisting of direct measurement of intact, fresh samples using infrared spectroscopy with no sample preparation could lead to accurate calibrations and will be investigated in the next section (Lopo et al., 2015; Musingarabwi et al., 2016; Diago et al., 2018; Fernández-Novales et al., 2019; Cuq et al., 2020; Jones et al., 2020).

### Direct Quantification Techniques Using Infrared Spectroscopy

The investigation of using infrared spectroscopy for the non-invasive measurement of fresh grapevine samples could lead to direct, in-field applications allowing for the implementation of precision viticulture. Although limited studies have been reported on fresh samples, the feasibility of using infrared spectroscopy as a direct, non-invasive quantification method for nitrogen, amino acids, and other metabolites, are explored in this section (Fernández-Novales et al., 2019; Cuq et al., 2020).

A method using on-the-go, contactless NIR explored the possibility of monitoring grapevine water status (Diago et al., 2018). Predictive models were compiled with R^2^ values ranging from 0.68 to 0.85. The models were used to spatially map the water status variability of a vineyard on different dates, providing useful information and facilitating decisions regarding irrigation schedules (Diago et al., 2018).

Vis-NIR reflectance spectroscopy was also used to explore quality attributes in intact grape berries (Dos Santos Costa et al., 2019; Fernández-Novales et al., 2019). Fernández-Novales et al. (2019) investigated amino acid and total soluble solid content using PLS with fivefold cross validation. Samples were collected from one cultivar and one vineyard with 128 grape clusters sampled at five phenological stages. Accurate models were developed for total soluble solids, but the amino acid calibration models were only sufficient for screening purposes (Fernández-Novales et al., 2019). The quantification of amino acid content of berries, even just for screening purposes, could greatly aid harvesting and oenological decisions. Dos Santos Costa et al. (2019) achieved robust prediction models for total soluble solids and total anthocyanins using 432 Shiraz and 576 Cabernet Sauvignon berries. Separate models for Shiraz and Cabernet Sauvignon berries were developed, as well as a combined model. Similar results were reported for the separate and combined models (Dos Santos Costa et al., 2019). These studies were conducted on limited cultivars and vineyards, and although a large number of samples were included the representativeness of those samples were restricted. Future research should include various cultivars, regions, grapevine organs, and phenological stages to include sufficient variability and representativeness in the dataset. The similar results found for separate models per cultivar and for combined models suggest that individualized models should be explored and could lead to more accurate prediction calibrations.

Lastly, a study conducted in southern France used NIR spectroscopy to investigate and assess macro-elements in fresh grapevine leaves and berries (Cuq et al., 2020). The macro elements that were considered in this study were carbon (C), hydrogen (H), nitrogen (N), and sulfur (S). Four cultivars were sampled across 63 plots, and leaf petioles, leaf blades, pea-size berries and véraison berries were collected for each plot leading to 252 total samples. Each sample was made up of a representative number of leaves (50) or berries (200). The blades and petioles of the same leaves, and berries at two phenological stages (pea-size and véraison) were measured as fresh and dried (homogenized) samples. PLS regression using test set validation was employed with a 75:25 split. During model development all data for leaves and berries were combined, but separate models for fresh and dried samples were compiled. Model performance showed only the models for nitrogen and carbon:nitrogen ratio was usable according to the residual predictive deviation (RPD) for fresh and dried samples. The models for the dried samples still performed somewhat better than the fresh samples (Cuq et al., 2020).

However, the predictive difference observed for fresh and dried samples could be offset by the time needed for sample preparation. Using fresh samples could also lead to direct, in-field applications. As previously discussed, this study found separation based on grapevine organs and phenological stage (Cuq et al., 2020). However, during model development, all the data was combined into a single database. Models for each organ were considered but the data was not shown, and the models were said to be inconclusive and less accurate than the combined data. The inconclusive results for the individual models could be due to limited samples, organs, or phenological stages in this study leading to poor representativeness in the dataset.

The studies discussed in this section investigated infrared spectroscopy for quantification of amino acids, quality parameters, and macro elements in fresh grapevine organs (Dos Santos Costa et al., 2019; Fernández-Novales et al., 2019; Cuq et al., 2020). Although the quantification of some key metabolites was reported in fresh grapevine organs, carbohydrate determination has not yet been attempted. The studies mostly focused on berries and although some reported sampling throughout the growing season, the data from all phenological stages were combined for model development. Similarly, when more than one grapevine organ was sampled, the data for all organs were combined into one dataset. The fact that grapevine organs are extremely heterogeneous, based on their morphological, anatomical, and chemical structure could lead to large variations between grapevine organs, and even within one organ at different phenological stages. This variation could explain why models that combine all the data from different organs, and phenological stages, into a single database for model development are not leading to very accurate prediction models.

By monitoring fresh grapevine organs at several phenological stages across the growing season, specific models for each organ, and possibly phenological stage, could be investigated. These individualized models could lead to more accurate predictions of complex chemical compounds such as carbohydrates, nitrogen, and amino acids. The successful monitoring of these key metabolites during the growing season could greatly aid the implementation of precision viticulture.

## Chemometric Applications

The investigation and interpretation of infrared spectra is a complex process. The MIR and NIR spectra show peaks and vibrations for all the major molecular bonds in the measured sample. Additionally, NIR also includes the combinations and overtones present (Massart, 1973; Massart et al., 1988; Varmuza and Filzmoser, 2016). Infrared spectroscopy generates spectral data that contains immense amounts of information. Therefore, as previously stated, infrared spectroscopy is seen as a fingerprinting technique because it provides information about several properties of a sample simultaneously (Cozzolino, 2014; Dos Santos et al., 2017).

Chemometrics is needed to decipher this information and is defined as the procedure of extracting relevant information from chemical data using mathematical and statistical tools (Massart, 1973; Massart et al., 1988; Varmuza and Filzmoser, 2016). Chemometrics, and more specifically multivariate data analysis (MDVA), has been extensively used for qualitative and quantitative applications in the agricultural industry (Dambergs et al., 2015; Varmuza and Filzmoser, 2016; Williams, 2019). Qualitative applications will be mentioned in this section, while quantitative chemometric applications will be discussed in more detail.

The chemometric techniques most often used in the literature discussed in this review for infrared spectroscopy data are PCA and PLS regression, as shown in Table 1. PCA is mostly utilized as a qualitative method for screening, grouping, extraction, and compression of multivariate data. Using mathematical procedures, the correlated response variables in spectral data are transformed into non-correlated variables known as principal components (PC’s) (Cozzolino et al., 2011; Dos Santos Costa et al., 2019; Cuq et al., 2020).

**TABLE 1:** Chemometric techniques and calibration parameters reported in discussed literature.

**References**	**Chemometric/MDVS technique used**	**Calibration parameters reported**
De Bei et al., 2017	PLS	R^2^_cal_, R^2^_val_, SECV, SEP, Slope, Bias, Rank^a^, RPD
Schmidtke et al., 2012	PLS and SVM	R^2^_val_, RMSEP, Bias, Rank
Jones et al., 2020	PLS	R^2^_cal_, R^2^_val_, RMSEC, RMSEV, RPD, Bias
Fernández-Novales et al., 2019	PLS	SEC, R^2^_cal_, SECV, R^2^_cv_, RPD, SEP, R^2^_pred_, Bias, Slope, Rank
Cuq et al., 2020	PLS, PCA	R^2^_cal_, RMSEC, RPD_cal_, R^2^_val_, RMSEV, RPD_val_, R^2^_pred_, RMSEP, RPD_pred_, percentage (reported for PCA)
Diago et al., 2018	PLS	R^2^_cal_, RMSEC, R^2^_cv_, RMSECV, R^2^_pred_, RMSEP
Dos Santos Costa et al., 2019	PCR, PLS, MLS for calibration models. PCA-LDA, PCA-QDA, PCA-LDA Mahalanobis, PLS-DA^b^ for maturation classification.	R^2^_cal_, RMSEC, SEC, R^2^_cv_, RMSECV, SECV, R^2^_pred_, RMSEP, SEP, Bias, Rank Percentage (reported for classification), confusion matrix
Petrovic et al., 2020	PLS	R^2^_cal_, RMSEC, RPD_cal_, R^2^_val_, RMSEV, RPD_val_, Slope, Bias, Rank
Aleixandre-Tudo et al., 2018a	PLS	R^2^_cal_, RMSECV, RPD_cal_, R^2^_val_, RMSEV, RPD_val_, Rank, ICC, SEM
Aleixandre-Tudo et al., 2018b	PLS	R^2^_cal_, RMSECV, R^2^_val_, RMSEV, RPD, Bias, Rank, Slope, ICC, SEM, LOD, LOQ
Wang et al., 2017	PCR, PLS, SMLR, BPNN^c^	R^2^_cal_, R^2^_val_, RMSEC, RMSEV
Quentin et al., 2017	PLS	R^2^_cal_, RMSEC, SEC, RPD_cal_, R^2^_pred_, RMSEP, RPD_pred_, Slope, Bias
Ramirez et al., 2015	PLS	R^2^_cal_, R^2^_cv_, R^2^_val_ RMSEC, RMSECV, RMSEP, RPD_cv_, RPD_val_, Rank

*^a^Rank defined as the number of latent variables or principal components/partial least square terms in calibration.*

*^b^PCA using linear discriminant analysis (PCA-LDA), quadratic discriminant analysis (PCA-QDA), linear discriminant analysis using Mahalanobis distance (PCA-LDA Mahalanobis) and PLS-DA.*

*^c^SMLR, stepwise multiple linear regression; BPNN, back propagation neural network.*

Although most literature in the agricultural and viticultural field report the use of PLS, it is worth mentioning that other regression techniques have recently been proposed that could outperform PLS. Ridge regression and lasso (least absolute shrinkage and selection operator) regression are variable selection methods that can be applied to spectroscopy data to reduce variables used during model development leading to more interpretable models (Piaskowski et al., 2016; Frizzarin et al., 2021; Gao et al., 2021). Although limited agricultural applications for ridge and lasso regression have been reported, it has been successfully employed to predict quality traits in cow’s milk (Frizzarin et al., 2021) as well as lignin content in poplar trees (Gao et al., 2021). However, in a study conducted on NIR data seven different regression methods were investigated including PLS, ridge, and lasso regression, to assess carbohydrates in wheat samples with PLS yielding the most accurate results (Piaskowski et al., 2016).

**FIGURE 2:**
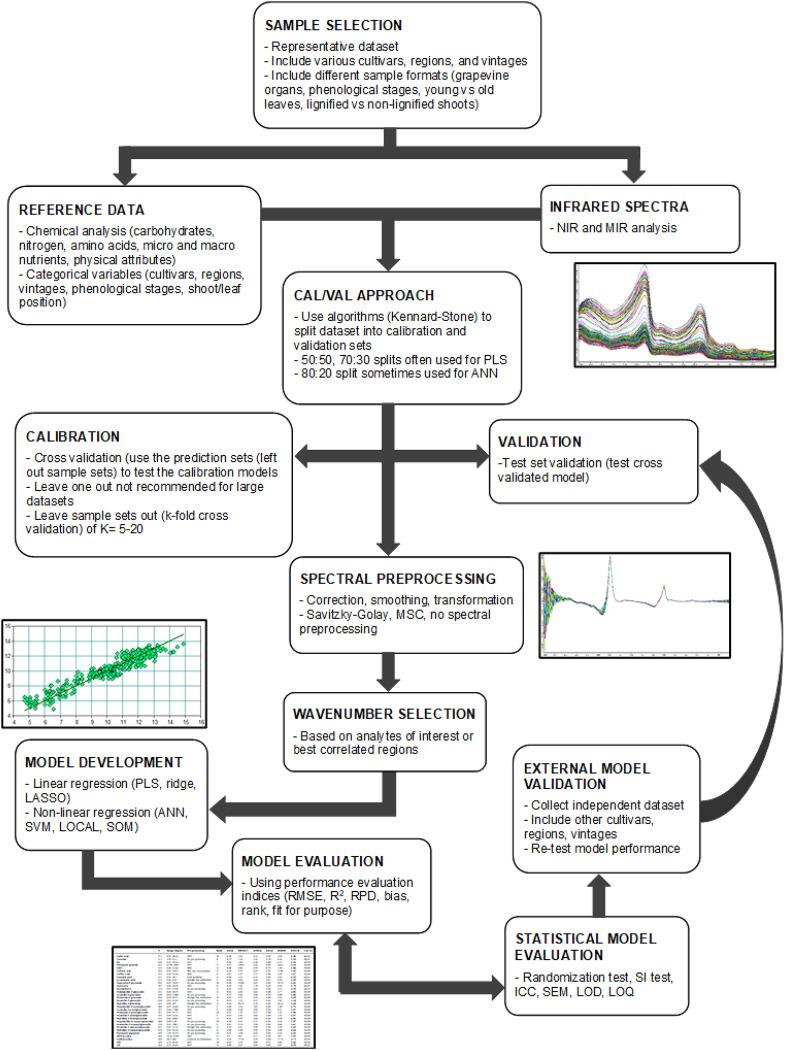
Regression workflow for model development using infrared spectra and grapevine reference data. NIR, near infrared; MIR, mid infrared; PLS, partial least squares; ANN, artificial neural networks; MSC, multiplicative scatter correction; LASSO, least absolute shrinkage and selection; SVM, support vector machines; LOCAL, locally weighted; SOM, Self-organizing maps; RMSE, root mean square error; RPD, residual predictive deviation; SI, slope and intercept; ICC, inter-class correlation coefficient; SEM, standard error of measurement; LOD, limit of detection; LOQ, limit of quantification.

Regression methods, such as PLS regression, are employed for classification purposes (PLS-DA) as well as quantification using spectral and reference data to compile prediction models. Chemometrics uses statistical techniques and reference data to extract and correlate the spectral data with a property of interest. This chemometric approach uses model development, including model calibration and validation, for the prediction and quantification of chemical and physical properties (Cozzolino et al., 2011; Schmidtke et al., 2012; De Bei et al., 2017; Bureau et al., 2019). Figure 2 shows the main steps during regression model development implemented on infrared spectra and grapevine reference data. Regression techniques employed for quantification purposes will be discussed extensively. Preprocessing techniques, model development steps, and performance evaluation indices and other methods used to compare model performance will be evaluated.

### Preprocessing Techniques

Before multivariate regression techniques are applied to spectral data, preprocessing is often used to enhance spectral features and remove undesirable sources of variation or irrelevant information (Dos Santos et al., 2017; Bureau et al., 2019). Various spectral preprocessing techniques exist, such as scattering or baseline correction, smoothing or transforming to first or second derivatives, standardization, and normalization. During model development some of the preprocessing methods are often investigated and the optimal technique or combination of techniques are chosen based on model performance (Nicolaï et al., 2007; Cozzolino et al., 2011; Varmuza and Filzmoser, 2016). As discussed in previous sections the study done by Cuq et al. (2020) was one of few that used fresh grapevine material. They employed preprocessing to eliminate light scattering effects and compensate for baseline bias and offset. They used multiple methods including the Savitzky-Golay algorithm and multiplicative scatter correction (MSC).

The Savitzky-Golay algorithm uses smoothing and transformation to remove random noise, baseline shifts and superimposed peaks to the first or second derivative. These derivatives help to highlight small peaks and bands and to elucidate overlapping peaks. The application of the MSC method to spectral data reduces the effect of light scattering and linearizes the spectra. MSC compensates for the non-uniform light scattering throughout the sample caused by particle size, refractive index, and radiation wavelengths (Nicolaï et al., 2007; Cozzolino et al., 2011; Varmuza and Filzmoser, 2016). Both these methods were proven to be useful when dealing with whole, fresh grapevine organs (Cuq et al., 2020).

Various other studies also used the Savitzky-Golay algorithm and MSC for preprocessing and these methods were often used in conjunction (Schmidtke et al., 2012; Dos Santos Costa et al., 2019; Fernández-Novales et al., 2019). Schmidtke et al. (2012) applied these algorithms to spectra from grapevine samples that were dried and ground to a powder, while Dos Santos Costa et al. (2019) and Fernández-Novales et al. (2019) used fresh grape berries. The wide application of these algorithms to dried and fresh samples show their applicability in the field of viticulture (Schmidtke et al., 2012; Dos Santos Costa et al., 2019; Fernández-Novales et al., 2019; Cuq et al., 2020).

The sample type, instrument, and purpose of the analysis all contribute to choosing an appropriate preprocessing technique (Stuart, 2004; Varmuza and Filzmoser, 2016; Türker-Kaya and Huck, 2017; Aleixandre-Tudo et al., 2019; Bureau et al., 2019). Often numerous preprocessing techniques and combinations need to be investigated to find the correct approach. The correct choice could also be not to implement any preprocessing techniques. The scattering components in some NIR spectra could include physical information of the sample such as density and removing scattering effects could reduce a sample’s physical information. Using smoothing techniques often only marginally improves the calibration model or even causes unfavorable effects. The use of smoothing could lead to decreased prediction accuracy because of correlations introduced in the noise structure (Olivieri, 2015). The option of using preprocessing or the raw spectra should be carefully considered before model development is initiated.

### Model Development

During linear regression model development, the dataset is often subdivided into a calibration set that is used to construct the model and a validation set used to test the model. This is done using various methods including test set validation, cross validation with leave-one-out or k-fold cross validation. During test set validation the dataset is split using algorithms, such as Kennard-Stone, based on a percentage (Porep et al., 2015; Petrovic et al., 2020). The split, between calibration and validation datasets, generally applied is 70:30 or 50:50 for PLS calibrations or even 80:20 for machine learning models. These dataset splits differ based on sample type and application (Fuentes et al., 2018; Bureau et al., 2019; Murru et al., 2019; Petrovic et al., 2020).

Alternatively, cross validation can be used. This involves leaving out one sample at a time for the construction of the calibration model, and then using the left-out sample for validation. However, with larger datasets it is often difficult to leave out one sample at a time because of the numerous calibrations generated when using this method and the minimal effect that leaving one sample out has on model performance. In the instance of large datasets, cross validation uses sample sets of up to ten or twenty samples that are left out at a time and used for validation (Cozzolino et al., 2011; Varmuza and Filzmoser, 2016). Similarly, when using k-fold cross validation the dataset is split into K subsets. One subset is applied for validation while the other K-1 subsets are used for calibration. This is repeated K times so that after K iterations all data is used for calibration as well as validation. K values between five and ten are commonly used (Paiva et al., 2021; Santos et al., 2021).

Although test set and cross validation datasets are considered as independent from a statistical viewpoint, model accuracy and robustness should preferably be tested with an independent, completely external sample test set. The samples for the external test set should be collected under the same conditions as the calibration set. Samples collected from different vintages, cultivars, and regions should be included in the external test set to assess the model’s robustness and ability to handle spectral variability in samples beyond those used in the calibration set (Cozzolino et al., 2011; Dos Santos et al., 2017; Bureau et al., 2019). Including sufficient variability could be difficult in agriculture and viticulture where ample variations exist between years because of vintage and climatic effects. However, if sufficient sample variability was included in previous vintages the model should have a better ability to predict future samples.

### Performance Evaluation Indices

The application of various preprocessing strategies, multivariate regression techniques and model optimizations lead to multiple calibration and validation models that need to be assessed based on their performance. Multiple performance evaluation indices or calibration parameters are used to report the results of calibration and validation models. The indices test the model’s accuracy and reliability (Aleixandre-Tudo et al., 2018b; Bureau et al., 2019; Dos Santos Costa et al., 2019; Fernández-Novales et al., 2019; Williams, 2019). The most commonly used parameters in the discussed literature are summarized in Table 1 and the values associated with them in Tables 2, 3.

**TABLE 2:** Direct and non-direct applications of infrared spectroscopy for quantification tasks in grapevines.

**References**	**Application**	**Sample type**	**Sample collection**	**IR technique**	**Chemometric/MDVS technique used**	**Calibration accuracy**
De Bei et al., 2017	Total non-structural carbohydrates (TNC), starch and sugar	Non-direct Trunk and leaf samples—Freeze dried, and ground to powder	One cultivar, two vintages, four phenological stages	NIR	PLS	**Trunk models:** TNC: R^2^_cal_ = 0.84, R^2^_val_ = 0.82, SECV = 10.92 mg/g, SEP = 12.65 mg/g, PC’s = 11, RPD = 2.17 Starch: R^2^_cal_ = 0.84, R^2^_val_ = 0.80, SECV = 10.60 mg/g, SEP = 12.51 mg/g, PC’s = 11, RPD = 2.51 **Leaf models:** TNC: R^2^_cal_ = 0.92, R^2^_val_ = 0.86, SECV = 17.83 mg/g, SEP = 22.86 mg/g, PC’s = 5, RPD = 3.48 Starch: R^2^_cal_ = 0.93, R^2^_val_ = 0.88, SECV = 16.18 mg/g, SEP = 21.13 mg/g, PC’s = 7, RPD = 3.73
Schmidtke et al., 2012	Nitrogen and starch reserves	Non-direct Root and wood samples—Freeze dried, and ground to powder	Three cultivars, four vintages, five regions (across two countries), 35 vineyards	ATR-FT-IR	PLS and SVM	**Nitrogen** SVM: R^2^ = 0,98, RMSEP = 0,07% DW PLS: R^2^ = 0,97, RMSEP = 0,08% DW **Starch** SVM: R^2^ = 0,95, RMSEP = 1,56% DW PLS: R^2^ = 0,95, RMSEP = 1,43% DW
Jones et al., 2020	Starch	Direct and non-direct Cane wood	Two cultivars, five vineyards, three vintages	NIR	PLS	**Starch -ground samples** R^2^ = 0.88, RMSEV = 0.73 mg/g, RPD = 2.85 **Starch—intact samples with bark** R^2^ = 0.20, RMSEV = 0.86 mg/g, RPD = 1.08 **Starch—intact samples without bark** R^2^ = 0.36, RMSEV = 0.75 mg/g, RPD = 1.24
Fernández-Novales et al., 2019	Amino acids	Direct Berries—fresh	One cultivar, one vineyard, five sampling dates	Vis + SW-NIR (Visible + Short wave NIR) and NIR	PLS	**Asparagine** SEC = 0.38 mg N/l, R^2^_cal_ = 0.71, SECV = 0.44 mg N/l, R^2^_cv_ = 0.64, RPD = 1.63, SEP = 0.45 mg N/l, R^2^_pred_ = 0.66
Cuq et al., 2020	C, H, N, S	Direct Leaves and berries—fresh	Four cultivars, 63 vineyards, two phenological stages	NIR	PLS, PCA	**Nitrogen** R^2^_cal_ = 0.90, RMSEC = 0.181% DW, RPD_cal_ = 3.14, R^2^_val_ = 0.84, RMSEV = 0.237% DW, RPD_val_ = 2.41, R^2^_pred_ = 0.91, RMSEP = 0.172% DW, RPD_pred_ = 3.32
Dos Santos Costa et al., 2019	Quality (total soluble solids, total anthocyanins, yellow flavonoids) and maturation	Direct Berries—fresh	Two cultivars, one region, one vintage	Vis/NIR	PCR, PLS, MLS for calibration models.	**Shiraz model** TSS: R^2^_cal_ = 0.97, RMSEC = 0.89%, SEC = 0.89%, R^2^_cv_ = 0.94, RMSECV = 1.33%, SECV = 1.33%, R^2^_pred_ = 0.95, RMSEP = 1.15%, SEP = 1.13%
					PCA-LDA, PCA-QDA, PLS-DA for maturation classification.	**Cabernet Sauvignon model** TSS: R^2^_cal_ = 0.97, RMSEC = 1.08%, SEC = 1.09%, R^2^_cv_ = 0.97, RMSECV = 1.13%, SECV = 1.13%, R^2^_pred_ = 0.96, RMSEP = 1.39%, SEP = 1.40%
						**Combined model** TSS: R^2^_cal_ = 0.96, RMSEC = 1.21%, SEC = 1.21%, R^2^_cv_ = 0.95, RMSECV = 1.37%, SECV = 1.37%, R^2^_pred_ = 0.95, RMSEP = 1.38%, SEP = 1.39%
						

**TABLE 3:** Direct and non-direct application of infrared spectroscopy for quantification tasks in other plants.

**References**	**Application**	**Sample type**	**Sample collection**	**IR technique**	**Model accuracy**
Veselá et al., 2007	Fat, nitrogen, and moisture	Cocoa powder	100 different brands	NIR and FTIR	**Nitrogen** NIR: R^2^ = 0.98, RMSECV = 1.7%, RMSEP = 0.09% FTIR: R^2^ = 0.95, RMSECV = 3.9%, RMSEP = 0.14% OPA with NIR and MIR: R^2^ = 0.96, RMSECV = 2.2%, RMSEP = 0.10%
Ramirez et al., 2015	Non-structural carbohydrates, sugar, and starch	Leaves and wood samples (including branch, stem, and root) – Dried and ground to powder	82 native tree species, four sites, two countries	NIR	**Total NSC (all tissues)** R^2^_cal_ = 0.93, R^2^_cv_ = 0.88, R^2^_val_ = 0.91, RMSEC = 1.12%, RMSECV = 1.43%, RMSEP = 1.34%, RPD_cv_ = 2.88, RPD_val_ = 3.26 **Total NSC (leaves)** R^2^_cal_ = 0.98, R^2^_cv_ = 0.94, R^2^_val_ = 0.68, RMSEC = 0.71%, RMSECV = 1.12%, RMSEP = 2.63%, RPD_cv_ = 4.22, RPD_val_ = 1.45 **Total NSC (stem and branches)** R^2^_cal_ = 0.97, R^2^_cv_ = 0.93, R^2^_val_ = 0.87, RMSEC = 0.67%, RMSECV = 1.00%, RMSEP = 1.22%, RPD_cv_ = 3.86, RPD_val_ = 2.58 **Total NSC (roots)** R^2^_cal_ = 0.99, R^2^_cv_ = 0.94, R^2^_val_ = 0.91, RMSEC = 0.55%, RMSECV = 1.11%, RMSEP = 1.18%, RPD_cv_ = 4.22, RPD_val_ = 3.41
Wang et al., 2017	Nitrogen	Leaves - fresh	One pear orchard, two vintages	Portable Vis/NIR	**Nitrogen** R^2^_cal_ = 0.86, R^2^_val_ = 0.81, RMSEC = 0.12, RMSEV = 0.13
Quentin et al., 2017	Non-structural carbohydrate, total soluble sugar, and starch	Leaves - Dried and ground to powder	24 Eucalyptus trees, over 8 months	NIR	**Total NSC** R^2^_cal_ = 0.88, RMSEC = 2.90%, SEC = 2.91%, RPD_cal_ = 2.79, R^2^_pred_ = 0.91, RMSEP = 2.35%, RPD_pred_ = 2.73
					

One of the most widely used parameters is the root mean square error (RMSE) that is used for calibration (RMSEC), cross validation (RMSECV) and prediction (RMSEP). RMSECV indicates the possible error for future predictions and RMSEP estimates the model’s ability to accurately predict new samples. Alternatively, the standard error of calibration (SEC), cross-validation (SECV) and standard error of prediction (SEP) could be used. RMSEP and SEP values are related but unlike RMSEP, SEP is independent of bias, while RMSEP include bias error. Some authors prefer using SEP together with bias while others favor RMSEP and some authors report all of the above as shown in Tables 1–3 (Aleixandre-Tudo et al., 2018b; Bureau et al., 2019; Dos Santos Costa et al., 2019; Fernández-Novales et al., 2019; Williams, 2019).

These parameters’ values are given in the same units as the measured compounds and should be as small as possible (Tables 2, 3). The threshold or accuracy will depend on the unit of measure for the compounds and the sensitivity of the existing analysis. The parameters can also be expressed as a percentage calculated using the population mean of the calibration or validation set used and a percentage below 20% is regarded as acceptable for analytical methods (Cozzolino et al., 2008; Torchio et al., 2013; Aleixandre-Tudo et al., 2019). These calculations do not include possible errors associated with the reference methods, but despite these limitations, they are still the most commonly used (Dos Santos et al., 2017).

Another performance index that is often used is the coefficient of determination or R squared (R^2^). R^2^ is used to explain the variance of the response variable in the calibration (R^2^_CAL_) and validation (R^2^_VAL_) sets. The value should be close to 1 so that as much variance as possible is explained for the response variable in the dataset (Cozzolino et al., 2011; Bureau et al., 2019).

The ratio of prediction to deviation or residual predictive deviation (RPD) is also often used to evaluate the predictive ability of a model (Ramirez et al., 2015; De Bei et al., 2017; Quentin et al., 2017; Cuq et al., 2020; Jones et al., 2020). RPD is calculated as the ratio of the standard deviation of the response variable to the RMSEP or RMSECV (Aleixandre-Tudo et al., 2019; Bureau et al., 2019). Values between 2 and 3 have been reported as acceptable for wine and grape applications (Aleixandre-Tudo et al., 2019; Cuq et al., 2020). In addition, other authors have interpreted values below 3 as adequate for screening and values above 5 capable of classification, quality, and process control tasks (Tables 2, 3) (Ramirez et al., 2015; De Bei et al., 2017; Quentin et al., 2017; Cuq et al., 2020; Jones et al., 2020). However, the RPD values’ interpretation is somewhat controversial since these thresholds were not determined using statistical basis (Cozzolino et al., 2011). Furthermore, other authors have argued that reporting both R^2^ and RPD is redundant since the calculation of RPD is inversely related to R squared (Minasny and McBratney, 2013).

Other statistics such as bias, rank and fit for purpose criterion are also used to assess model performance. Bias is measured as the difference between expected values (predicted) and true values (reference data) of a distribution. The selection of an optimal number of latent variable or principal components (often called rank) when using multivariate regression techniques such as PLS is also extremely important. If a model’s rank is too high there might be over-fitting and too low rank could lead to a model incapable of capturing all the variability present in the dataset (Cozzolino et al., 2011; Aleixandre-Tudo et al., 2019).

The fit for purpose criterion also needs to be considered after model development and judges the applicability of the model for routine use. The models need to be interpreted based on application as well as statistics (Cozzolino et al., 2011; Bureau et al., 2019). The successful implementation of a method also depends on model robustness. A calibration model is robust when the prediction accuracy is independent of external factors. Including a large representative sample set consisting of various cultivars, regions, vintages, and climatic conditions in the calibration model will help to ensure robustness (Cozzolino et al., 2011; Dos Santos et al., 2017; Petrovic et al., 2020).

In agricultural applications even less accurate prediction models can supply the ability to screen samples for low and high values. Rapid screening can be invaluable compared to the existing time-consuming and destructive methods. Although each of the performance evaluation indices do not supply all the answers, by investigating, and evaluating, all of them a clear picture of the regression techniques’ performance could emerge.

Tables 2, 3 list the studies using direct and indirect measurement of grapevines and other plants, respectively. The sample types, analytical application, and sampling procedures are reported together with the infrared and chemometric techniques used. Most of the studies reported numerous calibrations. However, only the most accurate calibration results were included in the tables. Most of the information shown in these tables were discussed in previous sections.

### Statistical Tests for Model Comparison

During model development with agricultural samples, a large number of models are often created to investigate the dataset. The values of the performance evaluation indices such as RMSE are often directly compared to evaluate the models’ predictive ability. Direct comparison of these absolute values can show differences between the models but not if the differences are significant in terms of model performance. Comparing the values directly might not be the best strategy to evaluate prediction performance. Statistical tests to compare the difference in observed and predicted values could be applied to evaluate significant differences (Olivieri, 2015; Aleixandre-Tudo et al., 2018a, 2019; Petrovic et al., 2020).

A randomization test has been investigated for the comparison of RMSE values. Hypothesis testing is employed to determine if calibrations differ significantly with the null hypothesis stating that the two compared RMSE values are equal (RMSE_1_ = RMSE_2_) and the alternative hypothesis proposing that one value is larger or different than the other (RMSE_1_ > RMSE_2_) (Van der Voet, 1994; Olivieri, 2015; Aleixandre-Tudo et al., 2019; Petrovic et al., 2020). Randomization testing could determine if significant differences between sample types, infrared methods or multivariate statistical techniques are present (Petrovic et al., 2020). Some literature has also suggested randomization testing for classification problems. Several classification methods could be compared, testing the hypothesis that two classification methods have similar classification ability (Van der Voet, 1994).

Other methods for model comparison, such as the slope and intercept test (SI test), have also been proposed in the literature (Linnet, 1993; Aleixandre-Tudo et al., 2018a, b). The SI test investigated the systematic error between the predicted values and the reference data and used a combined analysis of the regression line’s slope and intercept. SI testing can show if differences observed between predicted and reference values are due to random noise or not. The SI test can be used to evaluate and compare model performance and measurement methods such as infrared methods compared to reference methods (Linnet, 1993; Aleixandre-Tudo et al., 2018a, b).

Another proposed approach is the use of the inter-class correlation coefficient (ICC) and the standard “typical” error of measurement (SEM) calculated from the ICC. ICC is sensitive to detecting systematic error and both ICC and SEM are often used in reliability studies (Aleixandre-Tudo et al., 2018b). Reliability is defined as the consistency of measurements and ICC can be used to test the reliability of an instrument, person, or prediction value. The ICC values are unitless and are reported as a relative measure of reliability with values between 0 and 1. Values closer to 0 show no reliability and values closer to 1 indicate higher reliability. The magnitude of the ICC values depends on both the between sample variability as well as variability within the dataset (Yen and Lo, 2002; Weir, 2005; Aleixandre-Tudo et al., 2018a, b).

Therefore, the heterogeneity of the sample set should be considered when using ICC. Large ICC values could mask systematic errors when between-sample variability is high and low ICC values could still be found with a low systematic error and little between-sample variability. These values could lead to the conclusion that if samples are homogenous, it could be difficult to differentiate between them although the systematic error is small (Yen and Lo, 2002; Weir, 2005; Aleixandre-Tudo et al., 2018a, b).

The misinterpretation of the ICC values can be avoided by investigating the SEM in conjunction with ICC values. SEM values have the same unit as the measurement of interest and provide an absolute measure of reliability while ICC is a relative measure. SEM quantifies the precision of separate measurements and shows measurement error. SEM could also be used to construct confidence intervals for separate measurements and to determine the minimum difference needed to show true variance between separate measurements (Yen and Lo, 2002; Weir, 2005; Aleixandre-Tudo et al., 2018a, b).

Lastly, PLS calibrations could be assessed using the limit of detection (LOD) and limit of quantification (LOQ) adapted for multivariate purposes. LOD and LOQ values could still indicate the minimum concentration accurately predicted by the PLS calibration. Although, some studies have suggested that these values should be calculated for each sample. The multivariate LOD and LOQ will then be represented by a concentration range rather than a single value. LOD is seen as a good indicator of the quality of a calibration model because it incorporates the sensitivity and precision of analytical measurements (Allegrini and Olivieri, 2014; Olivieri, 2015; Aleixandre-Tudo et al., 2018b). Calibrations and methods could be evaluated and assessed using the statistical model comparison methods discussed. During model development, calibrations for each grapevine organ or developmental stage could be compiled and compared using the comparison methods.

### Non-linear Regression Techniques

The multivariate regression techniques discussed up to this point have all been linear regression methods, but sometimes agricultural models behave in a non-linear way. Linearity, or the lack thereof, can be assessed using statistical tests. Only when a statistically significant improvement of the prediction model is found, non-linear methods can be explored (Olivieri, 2015). Recently, non-linear regression techniques such as artificial neural networks (ANN) and Kernel-based techniques such as least squares support vector machines (LS-SVM) have been used more frequently (Fuentes et al., 2018; Murru et al., 2019).

Although ANN may perform better than linear techniques in some cases, the results are often difficult to understand, visualize, and interpret. Kernel-based techniques are often more favorable because they allow interpretation of the calibration model. LS-SVM has the benefit of including an added regularization parameter. The regularization parameter penalizes the use of large regression coefficient values leading to improved robustness of calibration models. Kernel versions of PCR and PLS as a logical extension of ordinary PCR and PLS have also been described. These statistical methods could be easier to work with for someone already familiar with PCR and PLS (Nicolaï et al., 2007; Cozzolino et al., 2011).

In recent viticultural research, non-linear methods have been employed for various classification purposes (Fuentes et al., 2018; Murru et al., 2019). Machine learning techniques such as ANN have been implemented together with NIR to develop models capable of cultivar classification based on leaf samples (Fuentes et al., 2018). FTIR and ANN have been utilized for the classification of grape samples according to cultivar and ripeness levels (Murru et al., 2019). LOCAL (locally weighted) regression has also been applied for the prediction of red grape quality parameters, and was found to perform better than PLS (Dambergs et al., 2006).

Other non-linear regression techniques, not widely used in the field of viticulture, should also be considered. Self-organizing maps (SOM) is a type of neural network specifically suited to large and multi-dimensional datasets making it ideal for spectral data (Tan et al., 2013; Wehrens and Kruisselbrink, 2018; Xu et al., 2019). SOM is an unsupervised clustering algorithm unique in that it transforms complex data into visually interpretable clusters and still preserve the topological properties of the input space (Milovanovic et al., 2019; Xu et al., 2019). This is achieved using a neighborhood function to plot objects into a two-dimensional space with similar objects close together and dissimilar objects further apart. SOMs can be implemented to assess clustering in data as well as investigate the structure within the clusters (Wehrens and Kruisselbrink, 2018; Milovanovic et al., 2019).

In a recent study PCA and SOM were used to investigate the classification of wine based on origin. Both methods yielded acceptable clustering results, although it was stated that SOM treatments provided better resolution. Although this study was not conducted on spectral data, the feasibility of using SOM as an alternative to PCA was shown. The added advantage of the unsupervised nature of SOMs is that no assumptions are made during clustering, visualization, and construction of the data (Milovanovic et al., 2019). The use of SOM in conjunction with PLS has also been proposed to improve spectral prediction models. SOM is initially used to cluster the data based on spectral variables or samples, after which PLS is performed on the clusters, leading to a consensus model. These models fully incorporate the sample information while highlighting the role of applicable variables and samples, and reducing the effect of useless variables or samples (Tan et al., 2013; Xu et al., 2019). Often a combination of linear and non-linear methods needs to be considered together with the technique’s purpose (prediction or classification) to achieve the desired outcome.

### Final Remarks

The main advantage of chemometric methods is that they look beyond the one-dimensional approach and investigate the sample in its entirety, making it well suited for use with spectroscopy. Both multivariate and infrared spectroscopy techniques do not assess just a single component but the interactions, interferences, and combined effects of the whole sample matrix. Many modern applications of spectroscopy techniques in the food industry are based on indirectly measuring chemical and physical properties. Instrumental techniques established using infrared spectroscopy are often correlated methods meaning that the measurement variable does not directly correlate with the compound of interest or the concentration of the compound (Gishen et al., 2005). The spectral regions identified in the prediction model might not correlate directly to the compound of interest. The complexity of spectra makes it difficult to correlate prediction models with the specific or relevant functional groups of a reference compound (Cozzolino, 2014). The correlation of the prediction models with other spectral regions can be explained by the fact that infrared spectroscopy measures all components in the sample, as well as the interaction between compounds, and the interference or combined effects they have on each other. Therefore, these interactions or interferences could contribute to the data used in the prediction model. Multivariate data analysis techniques can incorporate and investigate all the aspects of spectroscopy data making it the favored approach (Gishen et al., 2005).

Furthermore, new chemometric techniques and new ways of reporting results are continuously being developed. Recent studies have proposed using statistical tests such as the randomized test, SI test, ICC, SEM, LOD, and LOQ values to compare model performance (Allegrini and Olivieri, 2014; Olivieri, 2015; Aleixandre-Tudo et al., 2018a, b). Most studies still use direct comparison of the absolute values of the model performance indicators for the comparison of calibrations. Direct comparison is not the most suitable approach since it does not indicate if these values differ significantly, and if there truly are significant differences between the calibrations. The comparison methods could also be used for various purposes including testing the reliability of measurements, comparing instrumentation, detecting systematic error, and reporting the sensitivity of the calibration (Yen and Lo, 2002; Weir, 2005; Allegrini and Olivieri, 2014; Olivieri, 2015; Aleixandre-Tudo et al., 2018a, b). The future application of infrared spectroscopy will be elucidated in the following section.

## Discussion and Future Prospects

Despite numerous articles on infrared spectroscopy techniques published in scientific literature, the majority described feasibility studies (Dos Santos Costa et al., 2019; Fernández-Novales et al., 2019; Cuq et al., 2020; Jones et al., 2020). The heterogeneous nature of grapevine samples further complicates the matter, and a more representative sample set might be needed to accurately capture the variability, including various cultivars, regions, vintages, organs, and phenological stages. Thus, two contrasting approaches to optimize prediction models could be to construct a universal calibration or to build individualized calibrations per grapevine organ or phenological stage. Both these approaches will be discussed in this section.

However, the number of samples is not the most important factor, but rather how representative the samples are of future datasets. Studies conducted on just one cultivar or region could still measure thousands of samples without capturing the variability present in a vineyard (Schmidtke et al., 2012; De Bei et al., 2017; Rossouw et al., 2017; Fernández-Novales et al., 2019). During sample selection special care needs to be taken to incorporate representative samples. This could include sampling from various cultivars, regions, and vintages. Calibration development has also been mostly based on experimental data using cross-validation techniques where no independent sample set was used to validate the calibration model (Dambergs et al., 2015). Using a dataset of external and new samples to validate the calibration model could lead to more robust models capable of dealing with samples from different vintages, cultivars, or regions.

The frequent use of less representative sample sets of only one cultivar, organ, or phenological stage could be caused by expensive and time-consuming reference methods that are needed during calibration development. The reference methods require intense analytical, and human resources. Often cultivars, regions, vintages, organs, and phenological stages need to be analyzed to accurately capture the variability required for robust prediction models (Schmidtke et al., 2012; De Bei et al., 2017; Rossouw et al., 2017; Fernández-Novales et al., 2019).

In viticultural studies it is often difficult to include cultivars, regions, and vintages in a single study. Still, large variability of the samples is commonly needed to achieve sufficient robustness in the calibration (Schmidtke et al., 2012; De Bei et al., 2017; Dos Santos Costa et al., 2019; Fernández-Novales et al., 2019; Cuq et al., 2020). Similarly, conducting experiments on various climatic conditions, viticultural practices, or multiple growing regions could be challenging. It is not yet feasible to conduct studies including all these factors simultaneously. Therefore, collaborations among universities, research groups, and countries could be considered to develop a universal calibration capable of predicting multiple key metabolites in grapevines across the world. However, this will require global cooperation, immense datasets, and years to achieve.

Hyperspectral imaging (HSI) has also been proposed as a solution for the non-invasive quality assessment of fruits and vegetables. The main advantage of HSI is that it simultaneously provides the spatial and spectral information of the whole sample, whereas NIR provides the spectra of a given spot (Lorente et al., 2012; Chandrasekaran et al., 2019). The use of HSI for various quantification applications in grape berries has been reported, such as pH, anthocyanin, and soluble solid content (Chandrasekaran et al., 2019). A recent study done on grapevine bunches found that HSI was capable of distinguishing between healthy bunches and bunches infected with powdery mildew (Pérez-Roncal et al., 2020). Hyperspectral imaging has also been successfully applied to classification tasks such as the identification of grape varieties based on leaf spectra (Diago et al., 2013). The monitoring of vegetative indices throughout the growing season using HSI on grapevine leaves was also proposed (Yang et al., 2021). The use of NIR and MIR spectroscopy has been widely applied and researched for various agricultural and viticultural applications, unlike HSI. Although NIR and MIR spectroscopy generate extensive datasets, these datasets are still easier to analyze than the even more extensive datasets generated with HSI. While most research on HSI has been done in the last 2 years, these studies showed the significant potential of using HSI in future for non-invasive applications on grapevines.

Furthermore, studies have investigated portable infrared devices for in-field applications. Portable devices are mostly used for qualitative purposes such as classification (Gutiérrez et al., 2015; Lopo et al., 2015) while the samples for direct quantification are still taken to the laboratory (Fernández-Novales et al., 2019; Cuq et al., 2020). The few studies involving portable devices for viticultural applications have shown very contradicting results. Some recent studies have reported promising results for the prediction of water status in vineyards. The quantitative studies confirmed that NIR spectroscopy was robust and capable of reliably evaluating water status across diverse environmental conditions (Tardaguila et al., 2017; Diago et al., 2018). Although bench-top and portable devices represent different measurement technologies, similar measurement parameters were reported in a comparison study (Rodgers et al., 2017). However, portable devices often do not cover the entire spectral range and use fiber optics which could potentially introduce spectral noise (Reeves, 2010; Pasquini, 2018; Cuq et al., 2020). It has also been suggested that portable devices could lack the same quality components (detectors) found in bench-top instruments (Zumba et al., 2018).

Some studies have also raised the concern that extreme environmental conditions such as high temperatures or relative humidity could affect spectra (Zumba et al., 2018; Baca-Bocanegra et al., 2019). Temperature and humidity could affect samples’ moisture content and thus influence spectra (Zumba et al., 2018). A recent study investigated different detectors and sample temperatures under simulated field conditions. It was found that the temperature of the detector could influence spectral reproducibility, although sample temperature did not have the same effects. However, very few samples were included in the study (Phuphaphud et al., 2020).

The monitoring of grapevine organs throughout the growing season using infrared spectroscopy could yield valuable information. Extensive knowledge on the morphological and anatomical changes occurring in grapevine organs during the growing season are available, but the changes influencing spectral properties are not well understood. Changes have been observed in infrared spectra between different grapevine organs or phenological stages (Schmidtke et al., 2012; De Bei et al., 2017; Dos Santos Costa et al., 2019; Fernández-Novales et al., 2019; Cuq et al., 2020), but the reason for the differences have not been examined or explained.

Further investigation is needed to understand the relationship between morphological and anatomical changes, and spectral properties. These investigations could lead to the linking of certain morphological or anatomical changes to specific spectral properties. Spectral regions could be correlated to changes occurring throughout the growing season such as lignification or leaf aging. Once identified, the regions could be used for future monitoring of changes or used for classification purposes. The classifications could in turn lead to more individualized calibrations.

The heterogeneity found between grapevine organs and phenological stages has not often been considered during calibration development. Most calibrations have been compiled by combining all data from various organs and stages to yield one prediction model (Schmidtke et al., 2012; De Bei et al., 2017; Dos Santos Costa et al., 2019; Fernández-Novales et al., 2019; Cuq et al., 2020). Calibrations could be developed not just for each grapevine organ (shoots, leaves and berries), but possibly also for different phenological stages of the organs. Specific calibrations for young leaves vs. old leaves, or green shoots vs. lignified shoots could be established. This could possibly lead to more accurate and robust individualized calibration models, as an alternative to compiling a universal calibration.

The future of infrared spectroscopy lies in the rapid prediction of key metabolites in vineyards (Schmidtke et al., 2012; Rossouw et al., 2017; Cuq et al., 2020). Infrared technologies could lead to the continuous measurement and monitoring of the metabolites throughout the growing season under diverse conditions, which has not been feasible until now. There is still a lack of knowledge regarding the mobilization, accumulation, and storage of carbohydrates, nitrogen, and amino acids under different viticultural and climatic conditions. Infrared spectroscopy might supply the solution to monitor the metabolites on a continuous basis per vineyard block or even per grapevine. Continuous measurements could greatly increase our knowledge of the metabolites’ movement throughout the growing season under divergent conditions. This knowledge in turn could aid the implementation of precision viticulture and assist with viticultural decisions regarding fertilization, irrigation, and harvesting.

## Conclusion

Infrared spectroscopy has emerged as a rapid and reliable quantification method for agricultural crops. With the added benefit that infrared technology can be used for the direct measurement of intact plant material, it could lead to future agricultural applications. Infrared technologies could be implemented for the continuous monitoring of key metabolites in grapevine organs throughout the growing season. Continuous monitoring could greatly increase the knowledge of the movement and effects of these compounds under varying conditions.

The investigation of the grapevine organs’ spectral properties at different phenological stages could elucidate the changes occurring in the organs throughout the growing season. The observed spectral changes could lead to the classification of grapevine organs to develop individualized calibrations, possibly leading to improved quantification. Individualized calibrations based on grapevine organs or phenological stages could be considered to compensate for the heterogeneity in grapevines and develop more robust prediction models. The field of infrared spectroscopy is complex and comprehensive and could lead to specialized solutions for the broader agricultural sector, and more specifically the viticultural industry.

## Author Contributions

EW wrote the manuscript with support of JA-T, EB, and HN. EB and JA-T conceived the study, oversaw the overall direction, and planning and execution of the manuscript. EB, JA-T, and HN provided critical feedback on the manuscript. All authors helped shape the review manuscript.

## Conflict of Interest

The authors declare that the research was conducted in the absence of any commercial or financial relationships that could be construed as a potential conflict of interest.

## Publisher’s Note

All claims expressed in this article are solely those of the authors and do not necessarily represent those of their affiliated organizations, or those of the publisher, the editors and the reviewers. Any product that may be evaluated in this article, or claim that may be made by its manufacturer, is not guaranteed or endorsed by the publisher.
